# Postoperative Nausea and Vomiting in Patients Undergoing Laparoscopic Cholecystectomy under General Anaesthesia in a Tertiary Care Centre: A Descriptive Cross-sectional Study

**DOI:** 10.31729/jnma.7670

**Published:** 2022-09-30

**Authors:** Chitra Thapa, Gautam Ratna Bajracharya, Sulav Acharya, Nisha Shrestha

**Affiliations:** 1 Department of Anesthesiology, Nepal Medical College and Teaching Hospital, Jorpati, Kathmandu, Nepal

**Keywords:** *general anesthesia*, *laparoscopic cholecystectomy*, *postoperative nausea and vomiting*

## Abstract

**Introduction:**

Postoperative nausea and vomiting are frequent complications after laparoscopic cholecystectomy. Several risk factors have been associated with postoperative nausea and vomiting. This study aimed to find out the prevalence of postoperative nausea and vomiting in patients undergoing laparoscopic cholecystectomy under general anaesthesia in a tertiary care centre.

**Methods:**

A descriptive cross-sectional study was conducted among the patients undergoing laparoscopic cholecystectomy under general anaesthesia in a tertiary care centre from 1 July 2021 to 30 April 2022 after receiving ethical approval from the Institutional Review Committee (Reference number: 050-077/078). Convenience sampling was done. All the patients received antiemetic prophylaxis with ondansetron. The general anaesthetic technique was standardised in all the patients. They were followed up 24 hours after surgery for an episode of nausea and vomiting. Point estimate and 95% Confidence Interval were calculated.

**Results:**

Among 200 patients, postoperative nausea and vomiting were seen in 28 (14%) (9.19-18.81, 95% Confidence Interval). Among them, seven (25%) of the patients experienced post-operative vomiting as well.

**Conclusions:**

The prevalence of postoperative nausea and vomiting among patients undergoing laparoscopic cholecystectomy in our study was lower when compared to other studies conducted in similar settings.

## INTRODUCTION

Postoperative nausea and vomiting (PONV) include the undesired complications arising after anaesthesia in the post-anaesthesia care unit.^1,2^ Apfel proposed a risk score for PONV based on four factors: female gender, history of nausea and vomiting, abstinence from smoking and use of postoperative opioids. This score was found to produce satisfactory validation for clinical use.^3-7^

In our institute laparoscopic cholecystectomy is one of the most frequently performed surgical procedures. Ondansetron for PONV prophylaxis is administered to all patients undergoing laparoscopic cholecystectomy without risk assessment in this institute. However, to the best of our knowledge, the prevalence of PONV has not been studied in this setting to date.

This study aimed to find out the prevalence of postoperative nausea and vomiting in patients undergoing laparoscopic cholecystectomy under general anaesthesia in a tertiary care centre.

## METHODS

A descriptive cross-sectional study was conducted among the patients undergoing laparoscopic cholecystectomy under general anaesthesia at Nepal Medical College and Teaching Hospital from 1 July 2021 to 30 April 2022 after receiving ethical approval from the Institutional Review Committee (Reference number: 050-077/078). All the patients in the age group of 18-65 years with the American Society of Anesthesiologists (ASA) Physical Status Grade I or II who were scheduled for elective laparoscopic cholecystectomy under general anaesthesia at the hospital who provided consent for the study were included whereas, the patients taking antiemetic therapy, pregnant women, patients requiring management in Intensive Care Unit (ICU) and those unable to respond properly were excluded from the study. Convenience sampling was done. The sample size was calculated using the following formula:


$$\begin{array}{ll} \text{n} & ={\text{Z}}^{2}\times \frac{\text{p}\times \text{q}}{{\text{e}}^{\text{2}}}\\ & =1.96^{2}\times \frac{0.256\times 0.744}{0.07^{2}}\\ & =150 \end{array}$$

Where,

n= minimum required sample size
Z= 1.96 at 95% Confidence Interval (CI)
p= prevalence of PONV, 25.6%^8^
q= 1-p
e= margin of error, 7%

The minimum required sample size was 150. However, a total of 200 patients undergoing elective laparoscopic cholecystectomy were enrolled in this study. Preoperatively, a score was calculated based on the Apfel risk scoring system to find out the possibility of developing PONV. The score assigns one point each for the female gender, non-smoking status, history of PONV or motion sickness, and postoperative opioid use.^4^ All the patients received a similar standardised general anaesthetic technique. Intravenous paracetamol 15 mg/kg was given as preemptive analgesia. Thereafter, midazolam 20 μg/kg, fentanyl 2 μg/kg, propofol 1-2 mg/kg was given for induction, and rocuronium 0.9 mg/kg intravenously was given to facilitate the endotracheal intubation. Maintenance of anaesthesia was done with isoflurane in oxygen.

Ondansetron 4 mg was given to all the patients as antiemetic prophylaxis around half an hour prior to the completion of surgery. After removal of the gallbladder, intraperitoneal instillation of a 20 ml local anaesthetic agent was done by the surgeon. Residual neuromuscular blockade was reversed with a combination of neostigmine and glycopyrrolate as needed. After extubation patients were transferred to the post-anaesthesia care unit (PACU). The time of admission to the post-anaesthesia care unit marked the beginning of the 24-hour observation period. In the PACU, postoperative analgesia provided was paracetamol 1 gm 6 hourly and ketorolac 30 mg 8 hourly. PONV was assessed at 4-time points: at 2 hours, 4 hours, 6 hours and 24 hours.

Nausea was defined as a sensation of an urge to vomit, in the absence of expulsive muscular movements. Vomiting or emesis was defined as the forceful oral expulsion of the gastrointestinal contents. The episode where no gastric contents were expelled was labelled as retching. Retching and vomiting were collectively termed vomiting. PONV is typically used to describe nausea and/or vomiting or retching in the postanaesthesia care unit (PACU) or in the immediate 24 postoperative hours.^9^ The number of episodes of nausea and vomiting experienced in the first 24 hours was recorded.

Data were entered and analysed in IBM SPSS Statistics 16.0. Point estimate and 95% CI were calculated.

## RESULTS

**Figure 1:**
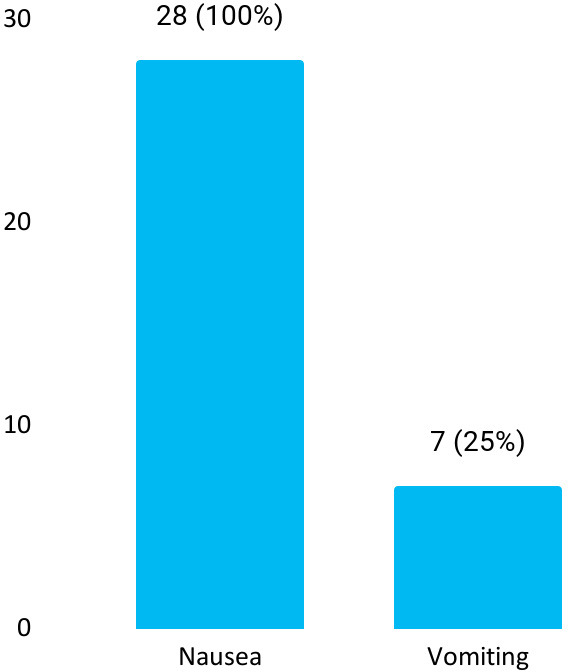
Patients experiencing nausea and vomiting (n= 28).

Among 200 patients, postoperative nausea and vomiting were seen in 28 (14%) (9.19-18.81, 95% CI). Seven (25%) patients experienced nausea as well as vomiting (Figure 1).

The average age of the patients with PONV was 40.64±12.06 years. Out of these patients, 28 (100%) were females. The ASA grading was I in 16 (57.14%) patients and II in 12 (42.85%) patients. After 6 and 24 hours of post-operative period, 18 (64.29%) and 1 (3.57%) patients had nausea and vomiting respectively (Table 1).

**Table 1:** Number of patients having nausea and vomiting in different hours (n= 28).

Postoperative period (hours)				
	2	4	6	24
**Nausea**	4	5	18	1 (3.57)
	(14.28)	(17.86)	(64.29)	
**Vomiting**	-	-	7 (25)	-

A history of PONV or motion sickness was present in 17 (60.71%) patients (Table 2).

**Table 2:** PONV in patients with different factors under Apfel score (n= 28).

Possible factors	Total n (%)	Nausea n (%)	Vomiting n (%)
Female gender	28 (100)	28 (100)	7 (25)
Non-smoker	26 (92.85)	26 (92.85)	7 (25)
History of PONV/motion sickness	17 (60.71)	17 (60.71)	3 (10.71)
Postoperative opioids	-	-	-

As for the presence of different factors described by Apfel, two (7.14%) had three factors, 17 (60.14%) had two factors and 11 (39.28%) had one factor (Table 3).

**Table 3:** Postoperative nausea and vomiting according to Apfel's score (n= 28).

Apfel's score	Number of individuals	Nausea n (%)	Vomiting n (%)
0	-	-	-
1	11 (39.28)	11 (39.28)	4 (14.28)
2	17 (60.14)	17 (60.14)	3 (10.71)
3	2 (7.14)	2 (7.14)	-
4	-		

## DISCUSSION

The prevalence of PONV was 14% among patients undergoing laparoscopic cholecystectomy in our study. This study was conducted only in patients undergoing laparoscopic cholecystectomy as laparoscopicsurgeries have been linked with a remarkably high rate of PONV and are therefore regarded as standard operations for the study of PONV.^10^ As per the consensus guidelines for managing postoperative nausea and vomiting, pharmacologic prophylaxis should be based on risk stratification.^11^ Because of the ease of administration we have been using ondansetron in all the patients irrespective of the risk factors present. With single drug prophylaxis, we found a PONV prevalence of 14% in our population.

A study reported a PONV prevalence of 33.5% in patients undergoing laparoscopic cholecystectomy which is higher than the prevalence in our population.^12^ The difference could be due to different anaesthetic techniques such as the use of nitrous oxide, opioids, duration of anaesthesia and the timing of administration of the prophylactic drug used. Another study among 244 patients found a cumulative incidence of 0.51 (0.45-0.57, 95% CI) in 24 hours with emetic episodes in 33.2% of the patients and nausea in 46.7% which was quite high.^13^ The higher prevalence may be due to the type of prophylactic drug used. In their study, most patients (83.2%) received 10 mg of metoclopramide as prophylaxis for PONV, which is considered ineffective to reduce PONV.^11,14^ Also the patients who were given dimenhydrinate as prophylaxis, did not receive the recommended dose.^11^

Consensus guidelines for the treatment of postoperative nausea and vomiting published by the American Society of Anesthesiologists (ASA) and the Society for Ambulatory Anaesthesia (SAM) recommend a 5-HT3 antagonist as a first-line drug.^11^ In our study all the patients were given ondansetron for PONV prophylaxis, as it is the most studied, cheap and easily available drug. We administered ondansetron around half an hour before the end of surgery which may be the reason for greater effectiveness.^15^ None of the patients in our study received postoperative opioids for pain management, so none of the patients had a risk score of 4 which may have resulted in a lower prevalence of PONV.

In this study all the patients who had PONV were females (100%). This finding corelates with that of Apfel as they found female sex to be the most specific predictor of PONV.^17^ After female sex, non smoking status prevailed in our patients who had PONV, as 26 patients (92.85%) were non smokers. Least prevalent risk factor in our study was a history of PONV or motion sickness as 17 patients (60.71%) who experienced PONV had a history of PONV or motion sickness. None of the patients who experienced PONV had an Apfel score of 0. Eleven patients who experienced PONV had an Apfel risk score of 1. Despite being a low risk group, these patients experienced PONV. Seventeen patients with an Apfel risk score of 2, experienced PONV in our study. Our single agent prophylaxis seems to be inadequate in these patients.

According to the consensus guidelines for the treatment of PONV, patients with moderate risk of PONV should receive prophylaxis with two or more drugs from different classes. Two patients with an Apfel risk score of 3 experienced PONV. This may be due to lesser number of patients in high risk group in our study. Though we found a lower PONV prevalence with single agent prophylaxis, a study when used PONV prophylaxis according to the risk score found a reduction of PONV from 29.9 to 9.8%.^18^

An association between the components of the Apfel risk score and the occurrence of PONV could not be made in this study because of the nature of the study design. Also, the small sample size and the single-centric nature of the study could limit the generalizability of the findings of this study as the study was conducted in a single tertiary care centre which might not depict the true picture of the patients all over the country.

## CONCLUSIONS

The prevalence of postoperative nausea and vomiting among patients undergoing laparoscopic cholecystectomy in our study was lower when compared to other studies conducted in similar settings. The prevalence of PONV in patients could be reduced further by offering prophylactic management based on risk stratification. For this, higher studies are warranted.
